# Effet du décubitus ventral vigile chez les patients non intubés atteints d’une pneumonie hypoxémiante

**DOI:** 10.48327/mtsi.v5i4.2025.772

**Published:** 2025-10-27

**Authors:** Karama BOUCHAALA, Mabrouk BAHLOUL, Sabrine BRADAI, Rania AMMAR, Chokri BEN HAMIDA

**Affiliations:** Service de réanimation médicale du Centre hospitalier universitaire (CHU) Habib Bourguiba, route EL Ain km 1. Code postal 3029. Sfax, Tunisie

**Keywords:** Insuffisance respiratoire aiguë, Détresse respiratoire aiguë, Covid-19, Décubitus ventral vigile, Pays en développement, Service de réanimation de Sfax, Tunisie, Acute Respiratory Failure, Acute Respiratory Distress, COVID-19, Awake prone positioning, Low-income country, Intensive care unit in Sfax, Tunisia

## Abstract

**Introduction:**

Plusieurs études ont suggéré que l’utilisation précoce du décubitus ventral vigile (DV) dans la prise en charge de l’insuffisance respiratoire aiguë due à une pneumopathie communautaire grave, stable sur le plan hémodynamique avec une vigilance normale, peut améliorer l’oxygénation et éviter le recours à une ventilation mécanique invasive. Cela peut également contribuer à réduire la létalité. Son bénéfice chez les patients oxygéno-dépendants hospitalisés pour une pneumonie à Covid-19 a été évalué chez des patients non intubés souffrant d’insuffisance respiratoire aiguë. Nous avons étudié à partir des données de la littérature, si l’application du DV pouvait améliorer l’hypoxémie et les signes d’insuffisance respiratoire aiguë chez les patients avec une pneumonie communautaire Covid ou non-Covid-19, diminuer le recours à la ventilation mécanique invasive et si son application précoce pouvait réduire la létalité chez les patients Covid-19.

**Matériel et méthode:**

Nous avons recherché sur Medline les articles publiés en français ou en anglais comportant les mots clefs « acute respiratory failure » ou « acute respiratory distress » et « prone position ».

**Résultats/Conclusion.:**

Le retournement en DV est une technique simple, non coûteuse et efficace qui permet d’améliorer le pronostic vital des patients souffrant d’une détresse respiratoire secondaire à une pneumonie communautaire grave, quel que soit l’agent causal. Cette technique est applicable facilement dans les pays à revenus faibles ou intermédiaires et dans les zones tropicales, en particulier Afrique du Nord, en Afrique subsaharienne, en Asie et en Amérique du Sud.

## Abstract (en)

**Introduction:**

Several studies have suggested that the early use of awake prone positioning (PP) in patients with acute respiratory failure due to severe community-acquired pneumonia, hemodynamically stable and alert, may improve oxygenation and avoid the need for invasive mechanical ventilation. PP may also help reduce case fatality rate (CFR). The benefits of PP for oxygen-dependent patients hospitalized with non-intubated acute respiratory failure due to SARS-CoV-2 infection have been evaluated. We reviewed the literature to determine if PP could improve hypoxemia and signs of acute respiratory failure in patients with community-acquired or non-community-acquired pneumonia, reduce the need for invasive mechanical ventilation, and reduce CFRin patients with Covid-19.

**Materials and methods:**

We searched with Medline for articles published in French or English containing the keywords “acute respiratory failure” or “acute respiratory distress” and “prone position.”

Results/Conclusion. Turning into prone position is a simple, inexpensive, and effective technique that improves the prognosis of patients with respiratory distress due to severe community-acquired pneumonia, regardless of the cause. This technique can be easily implemented in low-and middle-income countries, particularly in North Africa, sub-Saharan Africa, Asia, and South America.

## Introduction

Les pneumonies graves se manifestent par une détresse respiratoire aiguë et une hypoxémie, qui est la cause de décès la plus fréquente [2,5, 6,20,27,30,34]. Ainsi, lors de la pandémie de Covid-19, les unités de soins intensifs ont fait face en 2020-2021 à l’admission d’un grand nombre de patients présentant une insuffisance respiratoire aiguë allant d’une oxygénation à haut débit jusqu’au syndrome de détresse respiratoire aiguë (SDRA) et mettant en jeu le pronostic vital [30,31,35,37]. La ventilation mécanique invasive ou non était utilisée pour les cas les plus graves d’hypoxémie.

Le décubitus ventral (DV) chez un patient vigile en ventilation spontanée est une position dans laquelle une personne est allongée sur le ventre avec la tête tournée sur le côté.

Le retournement en décubitus ventral vigile est une technique simple recommandée dans le traitement des hypoxémies sévères. Peu coûteuse, elle nécessite des équipes formées et nombreuses [6]. Il est possible de l’appliquer en cas de détresse respiratoire secondaire à une pneumonie communautaire grave, quelle que soit son étiologie, chez des patients conscients et stables sur le plan hémodynamique [12,42]. Elle peut être utilisée aux urgences, dans les services de pneumologie et les unités de soins intensifs.

Lors de la pandémie de Covid-19, l’expérience de cette technique s’est considérablement élargie. Plusieurs études ont suggéré que son utilisation précoce pouvait améliorer l’état respiratoire [6,10] et réduire la létalité [1,8, 25].

En Afrique du Nord, comme dans différentes autres régions notamment dans les zones tropicales, les services de réanimation en charge des patients atteints de Covid-19 ont été ouverts aux patients graves, souffrant d’une détresse respiratoire. Dans ces services, le DV était souvent utilisé pour traiter les patients.

Il nous a paru intéressant de faire une revue de la littérature afin de voir si l’application du DV pouvait améliorer l’hypoxémie et les signes de détresse respiratoire, indépendamment de l’étiologie. D’autre part, nous avons recherché si l’application précoce du DV réduisait le recours à la ventilation mécanique invasive et la létalité, dans un contexte particulier, incluant l’afflux massif de patients et la réduction des ressources, sans perte de chance pour le patient.

## Méthodes

Nous avons recherché, sur Medline, les articles publiés en français ou en anglais comportant les mots clefs suivants: « *acute respiratory failure* » ou « *acute respiratory distress* » et «prone position* ». En raison d’une forte convergence de nombreuses études en faveur de l’utilisation de cette technique et l’absence apparente d’opinion inverse, nous avons opté pour une revue narrative. Nous n’avons inclus que les publications dont le texte intégral était disponible en anglais ou en français. Toutes les études présentées sous forme de résumé, celles dont le texte intégral n’était pas accessible et tous les cas cliniques ont été exclus. Nous avons privilégié les études prospectives randomisées, les méta-analyses et les revues systématiques de la littérature.

## Résultats

**Figure 1:**
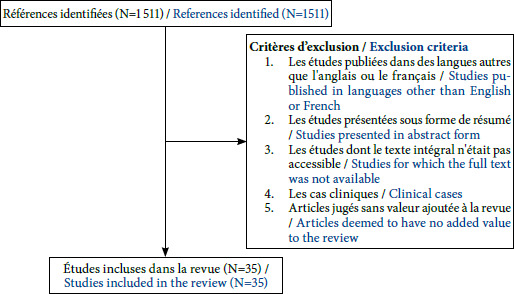
Diagramme de flux représentant le processus d’identification et de sélection des articles

À partir des mots clefs utilisés dans notre recherche, 1 511 articles ont été obtenus. Parmi ces derniers, le bénéfice du DV a été évalué dans la prise en charge de patients non intubés souffrant d>insuffisance respiratoire aiguë dans le cadre d’une pneumonie communautaire non-Covid-19 dans 2 études observationnelles [12,42], qui sont incluses dans notre analyse. Nous avons analysé 35 articles qui ont permis d’évaluer le bénéfice du DV dans la prise en charge de patients non intubés souffrant d’insuffisance respiratoire aiguë secondaire à une pneumonie à Covid-19. La figure 1 illustre le diagramme de flux représentant le processus d’identification et de sélection des articles [1,4,6,7,8,9,10,11-12,13-25,28,29,32,36,38,41,43,2,43,44,45,46,47,48,49-50].

## Discussion

Dans notre service de réanimation de Sfax (Tunisie) lors de la pandémie de Covid-19, nous avons opté pour la mise en DV de tous les patients conscients ayant une insuffisance respiratoire, à condition qu’ils soient vigiles, stables sur le plan hémodynamique, et adhèrent à la stratégie proposée.

La mise en décubitus ventral est perçue comme une technique complexe qui alourdit la charge de travail et comporte un risque élevé de complications. Cette technique est recommandée (grade IA) chez les patients atteints de SDRA avec hypoxémie sévère (rapport PaO_2_/FiO_2_<150) et nécessitant une ventilation mécanique [29]. Cependant, avant la pandémie de Covid-19, il n’existait aucune recommandation sur l’application du DV vigile chez les patients en détresse respiratoire conscients et en ventilation spontanée. Lors de cette pandémie, l’application du DV vigile a été utilisée en se basant sur des arguments physiologiques, physiopathologiques et par analogie au DV chez les patients atteints de SDRA sous ventilation mécanique [39].

Avant la pandémie de Covid-19, le DV chez les patients non intubés en insuffisance respiratoire aiguë a été appliqué uniquement dans 2 études observationnelles de 15 et 20 patients [12,42] qui rapportaient une amélioration de l’oxygénation et de la compliance du système respiratoire.

Plus récemment, des études chez les patients atteints de Covid-19 ont confirmé l’amélioration des échanges gazeux, la diminution du recours à la ventilation mécanique, de la mortalité et de la durée de séjour en réanimation et à l’hôpital [4,11,18,19,29,33,46,47,48,49,50,51-52].

**Figure 2:**
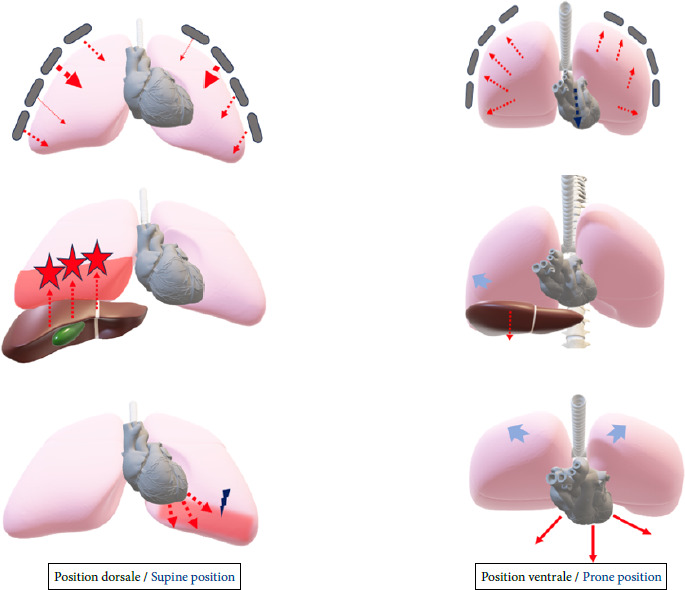
Comparaison de certains effets physiologiques du décubitus dorsal (gauche) et du décubitus ventral (droite) En décubitus ventral, les autres organes exercent moins de pression sur les poumons, ce qui améliore la compliance pulmonaire et le rapport ventilation-perfusion. En décubitus dorsal les flèches indiquent la direction de la pression exercée sur les poumons par la paroi thoracique, par le cœur et par les organes abdominaux. Le décubitus ventral a l’avantage de libérer le diaphragme postérieur contrairement au décubitus dorsal, ce qui améliore la compliance pulmonaire en réduisant l’effort nécessaire pour prendre de l’expansion et s’opposer à ces pressions. De plus, en décubitus dorsal le cœur va exercer une pression sur les deux bases pulmonaires qui sont déjà atteintes et va majorer les lésions préexistantes. De ce fait, l’application du DV va permettre d’éviter ce type d’aggravation secondaire à l’effet de masse qui s’exerce sur les deux poumons.

Le DV au cours du SRDA est utilisé pour améliorer la ventilation et l’oxygénation [39]. Lors du décubitus dorsal, ce sont les zones postérieures compressées (atélectasies) qui sont les mieux perfusées, ce qui aggrave le *shunt.* En position ventrale, les forces gravitationnelles exercées sur le parenchyme pulmonaire entraînent un meilleur recrutement des zones postérieures et permettent à une plus grande proportion d’alvéoles de participer aux échanges gazeux. De même, le DV a l’avantage de libérer le diaphragme postérieur contrairement au décubitus dorsal où il est déplacé vers le haut par les organes abdominaux, ce qui aggrave le collapsus pulmonaire postérieur [49]. Du point de vue mécanique, au cours du DV, l’expansion de la paroi thoracique antérieure se trouve limitée, ce qui permet une compliance thoraco-pulmonaire plus homogène et plus importante améliorant la ventilation des deux bases pulmonaires (Fig. 2). Le DV améliore la perfusion des zones pulmonaires bien aérées, ce qui augmente l’oxygénation en réduisant le déséquilibre du rapport ventilation/perfusion (V/Q) [49]. En améliorant la ventilation et l’oxygénation, le DV réduit le travail respiratoire du patient, ce qui peut être crucial en cas de troubles respiratoires sévères.

Le DV contribue à maintenir les voies aériennes postérieures ouvertes et favorise le drainage des sécrétions respiratoires. Enfin, en décubitus dorsal, le cœur comprime les deux bases pulmonaires, surtout à gauche, ce que le DV permet d’éviter [49]. Dans une étude locale, chez les patients pour lesquels nous avons obtenu un scanner thoracique en décubitus dorsal puis en DV, nous avons constaté une nette amélioration de la ventilation des deux bases pulmonaires [4].

Au total, beaucoup d’arguments physiopathologiques prédisent que l’application du DV vigile pourrait être bénéfique chez les patients en détresse respiratoire [7,8, 13,17,18]. Plusieurs études ont confirmé l’amélioration des échanges gazeux, la diminution du recours à la ventilation mécanique, et l’amélioration de la mécanique respiratoire suite à l’utilisation de cette technique [4,11,18,19,29,33,46].

Ces effets bénéfiques sur l’oxygénation ont été constatés dans plusieurs études [4,32,46-52]. Dans une étude locale, le DV a été associé à une amélioration du rapport PaO_2_/FiO_2_ durant les premières 24 heures [4]. Cependant, les jours suivants, cette amélioration ne s’était pas confirmée. Ceci pourrait être expliqué par les horaires des prélèvements sanguins qui coïncidaient avec le changement de position des patients pendant lequel les patients présentent souvent une désaturation artérielle en oxygène.

Dans une méta-analyse ayant recensé 16 études prospectives et rétrospectives et incluant 243 patients, Tan *et al.* ont montré l’efficacité du DV sur le rapport PaO_2_/FiO_2_ [47]. Ce rapport a été amélioré en moyenne de 47,9 (95% CI: 28,1-67,7; p<0,001). Cet effet bénéfique a été observé chez les patients ayant un SDRA secondaire à une infection par le virus SARS-CoV2 mais également due à d’autres étiologies [12,18,42]. Ce résultat a été retrouvé dans une méta-analyse plus récente incluant 14 études et 2 352 patients [18].

De même, l’amélioration de l’oxygénation a été confirmée par des études qui ont évalué la saturation pulsée en oxygène (SpO_2_) avant et après DV.

Dans notre étude, l’application du DV a été associée à une augmentation significative de la SpO_2_ qui est passée de 82 ± 11% à 96 ± 3% (p<0,001) [4]. Une élévation des chiffres de SpO_2_ lors du DV a été démontrée dans plusieurs études et méta-analyses [7,46]. Dans une méta-analyse, publiée par Tan *et al.* [47], regroupant 16 études et englobant 243 patients, la valeur de la SpO_2_ a significativement augmenté après la mise en DV (ΔSpO_2_ = 4,58; 95% CI: 1,35-7,80; p=0,005).

La tachypnée reflète la gravité de la détresse respiratoire et le travail imposé à l’appareil respiratoire. Les effets positifs du DV sur les échanges gazeux et l’amélioration de l’oxygénation suggèrent que le DV vigile permet une diminution du travail et de la fréquence respiratoires [25,46]. Dans notre étude, nous avons constaté que l’application de DV a permis une baisse significative de la fréquence respiratoire de 31±10 à 21±4 cycles/min (p<0,001), avec une nette régression des signes de lutte respiratoire [4]. La méta-analyse de Tan *et al.* confirmait une baisse significative de la fréquence respiratoire (ΔFR=-5,01; 95% CI: 8,49 -1,52; p=0,005) [47].

L’amélioration de l’oxygénation et la réduction du travail respiratoire au cours du DV évitent l’épuisement respiratoire du patient, retardent le recours à l’intubation et permettent parfois de l’éviter.

Dans une méta-analyse portant sur 5 530 patients recensés dans 32 études, le DV a diminué significativement le recours à la ventilation mécanique dans le groupe DV (+) comparativement au groupe DV (-) dans 6 études randomisées (n = 2 156; risque relatif (RR) = 0,81; 95% CI 0,720,90; p=0,0002) et 18 études non randomisées (n=3 374; RR=0,65; 95% CI 0,50-0,85; p=0,002) [28]. Cependant, dans une autre méta-analyse, publiée par Chua *et al.,* ayant regroupé 1 712 patients de 35 études differentes, les investigateurs n’ont pas constaté de différence significative concernant le recours à la ventilation mécanique (OR=1,20; 95% CI 0,77-1,86; p=0,42) [8], et ceci malgré l’amélioration significative de l’oxygénation dans le groupe DV (+). Cette différence pourrait être expliquée par la différence entre le nombre d’études et de patients inclus dans les deux méta-analyses.

**Figure 3:**
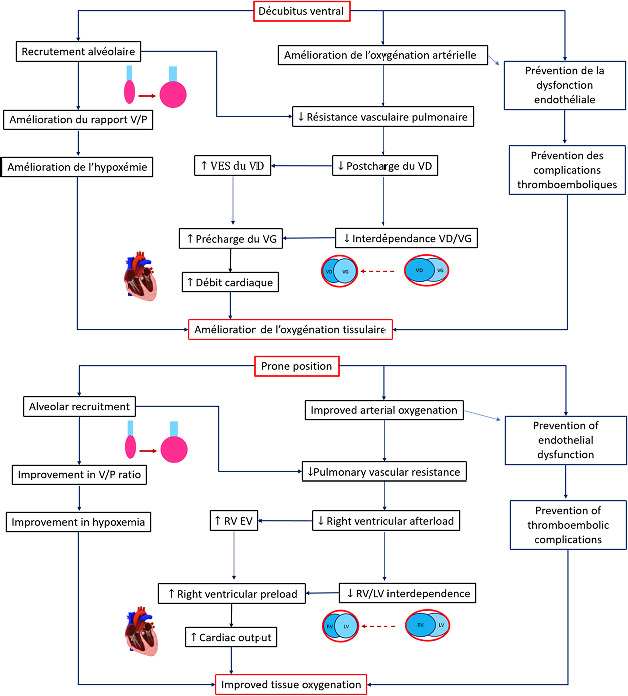
Effets hémodynamique et respiratoire du décubitus ventral (DV) L’application de la position ventrale entraîne un recrutement alvéolaire avec amélioration du rapport ventilation/perfusion (V/P). La conséquence est une amélioration de l’oxygénation artérielle avec baisse de la postcharge ventriculaire droite chez les patients atteints de SDRA, aboutissant à une amélioration de la fonction biventriculaire, et donc du débit cardiaque et de l’oxygénation cellulaire. Par ailleurs, l’amélioration de l’hypoxémie grâce à l’application du DV, permet de prévenir le dysfonctionnement endothélial et, par conséquent, des complications thrombo-emboliques. VES: volume d’éjection; VD: ventricule droit; VG: ventricule gauche

En dehors de ses effets bénéfiques sur la fonction respiratoire, le DV entraîne une amélioration de l’état hémodynamique, de l’oxygénation cellulaire et cérébrale:

- Sur le plan hémodynamique, il est bien établi que la position ventrale améliore la fonction cardiaque [3,26]. Elle entraîne une augmentation de la précharge cardiaque par augmentation du retour veineux, favorise la réduction de la vasoconstriction pulmonaire hypoxique et diminue la postcharge ventriculaire droite chez les patients atteints de SDRA [3,26]. Cela se traduit par une augmentation du débit cardiaque chez les patients disposant d’une réserve de précharge, avec des effets macro-circulatoires bénéfiques pour les organes nobles. Toutefois, en cas de pression intra-abdominale excessive, la survenue d’un collapsus complet de la veine cave inférieure, en particulier en cas d’hypovolémie, explique la fréquence de l’hypotension artérielle observée lors du retournement des patients.
- Le DV permet de prévenir la dysfonction endothéliale et la formation de thromboses entraînées par l’hypoxémie profonde [26]. L’amélioration de l’oxygénation grâce à l’application du DV permet de prévenir ce phénomène et, par conséquent, les complications thrombo-emboliques. Ces effets bénéfiques peuvent participer à l’amélioration pronostique de ces patients (Fig. 3).

Au cours de la Covid-19, le décès est essentiellement dû à une cause respiratoire (SDRA grave) ou aux complications de la ventilation mécanique [7,8, 13]. Les effets positifs du DV sur le plan gazométrique, la mécanique ventilatoire et le recours à l’intubation, réduisent la létalité [1,8, 16,40]. Dans notre étude [4], après 20 jours d’évolution, les patients en DV ont présenté un taux de survie estimé à 60% contre 20% chez les patients sans DV (p=0,59). Dans leur méta-analyse, Chua *et al.* ont montré que le groupe en DV avait présenté une létalité significativement inférieure au groupe avec décubitus dorsal (rapport de cotes (OR)=0,44; 95% CI 0,24-0,80; p=0,007) [8].

Cette diminution de la létalité a été confirmée dans une méta-analyse plus récente incluant 14 études et 2 352 patients [18]. La létalité a été significativement plus faible dans le groupe DV (+) *Vs* DV (-): 19,4% vs 26,8%; OR = 0. 51; 95% CI 0,32-0,80; p=0,003).

Notre analyse confirme que le DV a rarement été utilisé avant la pandémie de Covid-19 chez les patients ayant une insuffisance respiratoire aiguë due à une pneumopathie communautaire grave non intubés. Cette technique a été adoptée dès la première vague épidémique dans de nombreux pays sous-équipés et en situation de crise fortement consommatrice de moyens (lits et respirateurs de réanimation). Simple, peu coûteuse et efficace, elle peut être utilisée dans les pays à revenu faible et intermédiaire disposant d’infrastructures de santé limitées. Son application peut améliorer l’oxygénation, éviter le recours à une ventilation mécanique invasive et contribuer à réduire la létalité chez les patients ayant une insuffisance respiratoire aiguë d’origine infectieuse. Cependant, cette technique alourdit la charge de travail et comporte un risque élevé de complications pour les patients. En conséquence, elle nécessite des équipes formées et nombreuses. Une revue narrative peut présenter une limite méthodologique par rapport à une revue systématique, dont la démarche est plus rigoureuse. Cependant, notre approche synthétique était fondée sur un nombre réduit de références constituées principalement par des études prospectives randomisées, des méta-analyses et des revues systématiques de la littérature dont les conclusions sont robustes.

Le mot « Covid » n’a pas été utilisé dans notre recherche documentaire afin d’élargir nos conclusions à l’ensemble des patients souffrant d’insuffisance respiratoire aiguë. L’objectif de notre revue était de voir si l’application du DV pouvait améliorer l’hypoxémie et les signes de détresse respiratoire, indépendamment du statut Covid-19. Nous avons, toutefois, vérifié que l’ajout du mot « Covid » aux mots clefs utilisés dans notre revue de la littérature, n’impactait pas le nombre des références obtenues. En effet, la majorité des études ont été publiées pendant la pandémie de Covid-19.

## Conclusion

Plusieurs études et méta-analyses ont démontré que l’utilisation précoce du DV dans la prise en charge de l’insuffisance respiratoire aiguë peut améliorer l’oxygénation, éviter le recours à une ventilation mécanique invasive et contribuer à réduire la létalité. Il s’agit d’une technique peu coûteuse et efficace que l’on recommande dans le traitement des hypoxémies profondes chez des patients atteints de SDRA d’origine infectieuse, stables sur le plan hémodynamique avec une conscience normale. Il s’agit d’une technique qui peut être appliquée facilement dans les pays à revenus faibles ou intermédiaires, en particulier dans les zones tropicales.

## Financement

Aucun

## Contribution des auteurs et autrices

Conceptualisation: MB, KB

Bibliographie: MB, KB

Supervision: MB, CBH

Validation: MB, SB, KB, RA, CBH

Rédaction: MB, KB

Révision: MB, KB

## Déclaration de liens d’intérêt

Aucun lien d’intérêt n’a été déclaré.
