# Supplementary material for: Effect of awake prone positioning in non-intubated patients with community-acquired pneumonia complicated by hypoxemia
Source: Med Trop Sante Int. 2025 Oct 27;5(4):mtsi.v5i4.2025.772. [Article in French] doi: 10.48327/mtsi.v5i4.2025.772 (PMC12957996; doi:10.48327/mtsi.v5i4.2025.772)
Supplement: Related texts

# Research article: Effect of awake prone positioning in non-intubated patients with community-acquired pneumonia complicated by hypoxemia

## Introduction

Severe pneumonia is characterized by acute respiratory distress and hypoxemia, the latter of which is the leading cause of death [2,5,6,20,27,30,34]. During the 2020-2021 Covid-19 pandemic, intensive care units admitted a large number of patients with acute respiratory failure, ranging from high-flow oxygenation to acute respiratory distress syndrome (ARDS), a life-threatening condition [30,31,35,37]. The most severe cases of hypoxemia required invasive or non-invasive mechanical ventilation.

Prone position (PP) in a conscious patient on spontaneous ventilation is a position in which a person lies on their stomach with their head turned to one side.

Turning into awake prone position is a simple technique recommended for treating severe hypoxemia. It is inexpensive but requires trained teams [6]. It can be used in cases of respiratory distress due to severe community-acquired pneumonia, regardless of etiology, in conscious and hemodynamically stable patients [12,42]. It can be used in emergency rooms, pulmonology departments, and intensive care units. Experience with this technique has expanded considerably during the ongoing SARS-CoV-2 pandemic. Several studies have suggested that early use of PP may improve respiratory status [6,10] and reduce CFR [1,8,25].

In North Africa and various other areas, particularly in tropical regions, intensive care units were opened to treat critically ill patients with respiratory distress due to Covid-19. In these units, PP was often used to treat patients.

We decided to review the literature to determine whether PP could improve hypoxemia and signs of respiratory distress regardless of etiology. Additionally, we investigated whether the early application of PP could reduce the need for invasive mechanical ventilation and CFR in a specific context, including a massive influx of patients and limited resources, without compromising patient outcomes.

## Methods

We searched Medline for articles published in French or English that contained the following keywords: “acute respiratory failure” or “acute respiratory distress” and “prone position”. Due to the strong consensus in favor of this technique and the apparent absence of opposing opinions, we opted for a narrative review. We only included publications whose full text was available in English or French. We excluded all studies presented in abstract form, those whose full text was inaccessible, and all clinical cases. We prioritized prospective randomized studies, meta-analyses, and systematic reviews of the literature.

## Results

Using the keywords in our search, we found 1,511 articles. Among these, two observational studies [12,42] evaluated the benefit of PP in the management of non-intubated patients with acute respiratory failure due to non-Covid-19 community-acquired pneumonia, and these studies are included in our analysis. We analyzed 35 articles evaluating the benefits of PP in managing non-intubated patients with acute respiratory failure due to Covid-19 community-acquired pneumonia. Figure 1 shows the flowchart representing the process of identifying and selecting articles [1,4,6,7-12,13-25,28,29,32,36,38,41,43-50].

**Figure 1:** Flow-chart of the articles identification and selection process

## Discussion

During the Covid-19 pandemic in our intensive care unit in Sfax, Tunisia, we opted to place all conscious patients with respiratory failure in PP, provided they were alert, hemodynamically stable, and adhered to the proposed strategy.

Prone positioning can be perceived as a complex technique that increases the workload and carries a high risk of complications. It is recommended (grade IA) for patients with acute respiratory distress syndrome (ARDS) and severe hypoxemia (PaO_2_/FiO_2_ ratio <150) who require mechanical ventilation [29]. Prior to the pandemic, however, there were no recommendations for using PP with conscious patients who were spontaneously breathing and experiencing respiratory distress.

During the pandemic, PP has been used based on physiological and pathophysiological arguments, as well as by analogy with its use in ARDS patients on mechanical ventilation [39].

Before the pandemic, PP in non-intubated patients with acute respiratory failure had only been studied in two observational trials involving 15 and 20 patients, respectively, which reported improved oxygenation and respiratory system compliance [12,42].

More recently, studies of patients with SARS-CoV-2 have confirmed improved gas exchange, reduced need for mechanical ventilation, lower lethality rates, and shorter intensive care and hospital stays [4,11,18,19,29, 33,46,47,48,49,50,51-52].

PP is used during ARDS to improve ventilation and oxygenation [39]. In the supine position, the posterior areas are best perfused (atelectasis), which aggravates the shunt. In the prone position, gravitational forces act on the lung parenchyma, leading to better recruitment of the posterior areas and enabling a greater proportion of alveoli to participate in gas exchange. Similarly, PP frees the posterior diaphragm while in the supine position, the posterior diaphragm is displaced upward by the abdominal organs, which aggravates posterior lung collapse [49]. From a mechanical point of view, PP limits the expansion of the anterior chest wall, allowing for greater and more homogeneous thoraco-pulmonary compliance and improving ventilation of both lung bases (Fig. 2). PP improves the perfusion of well-aerated lung areas, increasing oxygenation by reducing the imbalance in the ventilation/perfusion (V/Q) ratio [49]. PP reduces the patient’s respiratory effort by improving ventilation and oxygenation, which can be crucial in cases of severe respiratory disorders. PP helps keep posterior airways open and promotes drainage of respiratory secretions. In the supine position, the heart compresses both lung bases, especially the left. PP helps avoid this compression [49]. In a local study in which we obtained chest CT scans of patients in both the supine and PP positions, we observed a clear improvement in ventilation of both lung bases [4]. Overall, many pathophysiological arguments suggest that awake prone positioning could benefit patients with respiratory distress [7,8,13,17,18]. Several studies have confirmed improved gas exchange, reduced mechanical ventilation use, and improved respiratory mechanics following this technique’s use [4,11,18,19,29,33,46].

**Figure 2:** Comparison of the physiological effects of the supine (left) and prone positions (right) In the prone position, other organs exert less pressure on the lungs. This improves lung compliance and the ventilation-perfusion ratio. The arrows in the supine position indicate the direction of pressure exerted on the lungs by the chest wall, heart, and abdominal organs. Unlike the supine position, the prone position frees the posterior diaphragm, which improves lung compliance by reducing the effort required to expand and resist these pressures. Additionally, in the supine position, the heart exerts pressure on both lung bases, which are already affected, thereby increasing pre-existing lesions. Therefore, using PP will prevent this type of secondary aggravation due to the mass effect exerted on both lungs.

These beneficial effects on oxygenation have been observed in several studies [4,32,46,47,48,49,50,51-52].

In a local study, PP was associated with an improvement in the PaO_2_/FiO_2_ ratio during the first 24 hours [4]. However, this improvement was not confirmed in subsequent days. This could be explained by the timing of blood sampling, which coincided with repositioning patients. During this process, patients often experience arterial oxygen desaturation.

Tan *et al.* demonstrated the effectiveness of PP on the PaO2/FiO2 ratio in a meta-analysis of 16 prospective and retrospective studies involving 243 patients [47]. The ratio improved by an average of 47.9 (95% CI: 28.1-67.7; p < 0.001). This beneficial effect was observed in patients with ARDS due to SARS-CoV2 infection as well as in patients with other etiologies [12,18,42]. A more recent meta-analysis including 14 studies and 2,352 patients found the same result [18]. Similarly, studies that assessed pulse oxygen saturation (SpO2) before and after PP confirmed the improvement in oxygenation. In our study, PP application was associated with a significant increase in SpO2, rising from 82 ± 11% to 96 ± 3% (p < 0.001) [4]. Several studies and meta-analyses have demonstrated an increase in SpO2 values during PP [7,46]. A meta-analysis by Tan *et al.* [47] included 16 studies and 243 patients and found that SpO2 values increased significantly after PP (ΔSpO2 = 4.58; 95% CI: 1.35-7.80; p = 0.005). Tachypnea reflects the severity of respiratory distress and the workload imposed on the respiratory system. The positive effects of PP on gas exchange and oxygenation suggest that it reduces respiratory workload and rate [25,46]. In our study, we found that PP use resulted in a significant respiratory rate decrease from 31 ± 10 to 21 ± 4 cycles per minute (p < 0.001) and a clear regression of respiratory distress signs [4]. A meta-analysis by Tan et al. confirmed a significant decrease in respiratory rate (ΔRR = -5.01; 95% CI: 8.49, -1.52; p = 0.005) [47].

Improved oxygenation and reduced respiratory effort during PP prevent respiratory exhaustion, delay the need for intubation, and sometimes render it unnecessary.

A meta-analysis of 5,530 patients from 32 studies revealed that PP significantly reduced the need for mechanical ventilation [28] in the PP (+) group compared to the PP (-) group. This reduction was observed in six randomized studies (n = 2,156; relative risk (RR) = 0.81; 95% confidence interval (CI): 0.72-0.90; p = 0.0002) and 18 non-randomized studies (n = 3,374; RR = 0.65; 95% CI: 0.50-0.85; p = 0.002) [28]. However, another meta-analysis by Chua *et al.* including 1,712 patients from 35 studies found no significant difference in mechanical ventilation use (OR = 1.20; 95% CI 0.77-1.86; p = 0.42) [8], despite significant oxygenation improvement in the PP (+) group. This discrepancy may be explained by the difference in the number of studies and patients included in the two meta-analyses.

In addition to its beneficial effects on respiratory function, PP improves hemodynamic status, as well as cellular and cerebral oxygenation:

- In terms of hemodynamics, it is well established that the prone position improves cardiac function [3,26]. It increases cardiac preload by enhancing venous return, reduces hypoxic pulmonary vasoconstriction, and decreases right ventricular afterload in patients with ARDS [3,26]. These changes result in an increase in cardiac output in patients with preload reserve and have beneficial macro-circulatory effects on vital organs. However, excessive intra-abdominal pressure can cause complete collapse of the inferior vena cava, especially in cases of hypovolemia. This explains the frequency of arterial hypotension observed when patients are turned over.
- PP prevents endothelial dysfunction and thrombosis caused by severe hypoxemia [26]. Improved oxygenation through PP prevents this phenomenon and consequently reduces the risk of thromboembolic complications. These beneficial effects may contribute to an improved prognosis for these patients (Fig. 3).

**Figure 3:** Hemodynamic and respiratory effects of prone position (PP) The application of the prone position leads to alveolar recruitment with improvement in the ventilation/perfusion (V/P) ratio. The result is improved arterial oxygenation with a decrease in right ventricular afterload in patients with ARDS, leading to improved biventricular function and, therefore, cardiac output and cellular oxygenation. In addition, the improvement in hypoxemia achieved by applying PP helps prevent endothelial dysfunction and, consequently, thromboembolic complications. EV: ejection volume; RV: right ventricle, LV: left ventricle

During the Covid-19, deaths are primarily due to respiratory causes, such as ARDS, or

complications from mechanical ventilation [7,8,13]. Positive effects of PP on blood gas levels, ventilatory mechanics, and the need for intubation, reduce CFR [1,8,16,40]. In our study [4], after 20 days of follow-up, the estimated survival rate was 60% for patients on PP compared to 20% for patients not on PP (p=0.59). In their meta-analysis, Chua *et al.* demonstrated that the PP group had a significantly lower CFR than the supine group (OR = 0.44; 95% CI= 0.24-0.80; p = 0.007) [8].

This reduction in CFR was confirmed in a more recent meta-analysis that included 14 studies and 2,352 patients [18]. CFR was significantly lower in the PP (+) group than in the PP (-) group (19.4% *vs.* 26.8%; OR = 0.51; 95% CI: 0.32-0.80; p = 0.003).

Our analysis confirms that the PP technique was rarely used before the start of the pandemic of Covid-19 on patients with acute respiratory failure due to severe community-acquired pneumonia and not intubated. However, from the first vague of the epidemic, many under-equipped countries adopted this technique. Simple, inexpensive, and effective, PP can be used in low-and middle-income countries with limited health infrastructures. It can improve oxygenation, avoid the need for invasive mechanical ventilation, and reduce CFR in patients with acute respiratory failure of infectious origin. However, this technique increases the workload and carries a high risk of complications for patients. Consequently, it requires large teams of trained personnel. Compared to a systematic review, a narrative review may present a methodological limitation due to its less rigorous approach. However, our synthetic approach was based on a small number of references, mainly consisting of randomized prospective studies, meta-analyses, and systematic reviews of literature whose conclusions are robust. We did not use the term “Covid” in our literature search in order to apply our conclusions to all patients with acute respiratory failure. Our review aimed to determine whether PP application could improve hypoxemia and signs of respiratory distress regardless of the patient’s Covid-19 status. Nevertheless, we found that adding the term “Covid” to the keywords in our literature review did not affect the number of references obtained. In fact, most of the studies were published during the Covid-19 pandemic.

## Conclusion

Several studies and meta-analyses have shown that early use of PP in the management of acute respiratory failure can improve oxygenation, avoid the need for invasive mechanical ventilation, and help reduce CFR. It is an inexpensive and effective technique that is recommended for the treatment of severe hypoxemia in patients with infectious ARDS, hemodynamically stable and with normal consciousness. It is a technique that can be easily applied in low-and middle-income countries, particularly in tropical areas.

## Funding

None

## Authors’ contributions

Conceptualization: MB, KB Bibliography: MB, KB Supervision: MB, CBH Validation: MB, SB, KB, RA, CBH Writing: MB, KB Revision: MB, KB

## Conflicts of interest

The authors declare that they have no conflicts of interest.
